# The effect of early pregnancy following chemotherapy on disease relapse and foetal outcome in women treated for gestational trophoblastic tumours

**DOI:** 10.1038/sj.bjc.6600041

**Published:** 2002-01-07

**Authors:** S P Blagden, M A Foskett, R A Fisher, D Short, S Fuller, E S Newlands, M J Seckl

**Affiliations:** Department of Medical Oncology, Charing Cross Hospital, London W6 8RF, UK

**Keywords:** trophoblastic disease, tumour, chemotherapy, pregnancy choriocarcinoma, relapse

## Abstract

Little literature exists on the safety of early pregnancy following chemotherapy. Here we assess the rate of relapse and foetal outcome in women who have completed single and multi-agent chemotherapy for gestational trophoblastic tumours. The records of 1532 patients treated for persistent gestational trophoblastic tumours at Charing Cross Hospital between 1969 and 1998 were reviewed. Patients were defined as receiving single agent or multi-agent treatment. Relapse rates and foetal outcome were reviewed in the 230 patients who became pregnant within 12 months of completing chemotherapy. In the single agent group 153 (22%) of 691 patients conceived early. Three subsequently relapsed. In the multi-agent group, 77 (10%) of 779 patients conceived early, two then relapsed. Relapse rates were 2% (3 out of 153) and 2.5% (2 out of 77) for each group compared to 5% and 5.6% in the comparative non-pregnant groups. Outcomes of 230 early pregnancies: 164 (71%) delivered at full term, 35 (15%) terminations, 26 (11%) spontaneous abortions, three (1.3%) new hydatidiform moles and two (1%) stillbirths. Early pregnancies were more common in the single agent group (*P*<0.001), but spontaneous miscarriages and terminations were more likely to occur in the multi-agent group (*P*=0.04 and 0.03, respectively). Of the full-term pregnancies, three (1.8%) babies were born with congenital abnormalities. Patients in either group who conceive within 12 months of completing chemotherapy are not at increased risk of relapse. Though, we still advise avoiding pregnancy within 12 months of completing chemotherapy, those that do conceive can be reassured of a likely favourable outcome.

*British Journal of Cancer* (2002) **86**, 26–30. DOI: 10.1038/sj/bjc/6600041 www.bjcancer.com

© 2002 The Cancer Research Campaign

## 

Cancers in young women including gestational trophoblastic tumours (GTT) (Seckl and Newlands, 1997), lymphomas (Vose *et al*, 1988), leukaemias (Rai *et al*, 1981) and ovarian germ cell tumours (Williams, 1996) are frequently cured with chemotherapy. Fertility is usually preserved following chemotherapy in these women (Berkowitz *et al*, 1998; Woolas *et al*, 1998), but little is known about the risk of disease relapse or damage to the foetus in women who conceive soon after completing treatment (Rustin *et al*, 1984). Many physicians advise their patients to avoid pregnancy during the first year of follow-up. It is thought that this allows ova which have been damaged by chemotherapy to either repair or undergo degeneration, reducing the risk of foetal malformation and/or spontaneous abortion. The greatest risk of tumour relapse is within the first year of remission and early pregnancy can compromise both the surveillance systems used to detect relapse as well as the safe institution of subsequent therapies. In addition, it is possible that the hormonal changes associated with pregnancy could in some instances actually promote tumour growth and early recurrence.

Patients with GTT provide a very good example of these difficulties. Nearly all women with the disease are cured with either single agent or combination drug chemotherapy (Newlands *et al*, 1986). Fertility is usually preserved and the affected women frequently wish to become pregnant again as soon as possible. This is often because they were keen to start or continue their family when they developed their original tumour. However, they are routinely advised to avoid pregnancy for at least 1 year post chemotherapy because: (1) most relapses occur in the first year post-treatment and these relapses are detected by a rising hCG secreted by the tumour cells (a normal pregnancy also produces hCG and this acts as a smoke screen masking the detection of tumour relapse); (2) the potential risk of cytotoxic drug induced damage to the ova (Sieber and Adamson, 1975; Schilsky *et al*, 1980; Choo *et al*, 1985); (3) the potentially increased risk of pregnancy induced relapse.

The advice to avoid pregnancy for 1 year post-chemotherapy in women with GTT has been determined by theoretical risks rather than solid clinical evidence of risk to the mother and foetus. Consequently, women who do become pregnant during this period and their physicians are faced with a dilemma of whether to continue or terminate the pregnancy (Kohorn, 1999). Though there is increasing published evidence that early pregnancy after GTT chemotherapy as a whole does not compromise the foetus (Song *et al*, 1988; Tuncer *et al*, 1999a,b; Berkowitz *et al*, 2000) there is little data regarding the effect of early pregnancy on subsequent disease relapse in the patient. Few have assessed these risks separately for each treatment group, i.e. those receiving single or multiple-agent treatment. Here we reviewed the number of relapses and the maternal and foetal outcomes in 230 women with GTT who became pregnant within 12 months of completing single or multi-agent chemotherapy. These results were compared with the number of relapses in women who did not become pregnant during the first 12 months after treatment for GTT and with national statistics on foetal outcome in unaffected healthy women.

## PATIENTS AND METHODS

We retrospectively reviewed the records of 1532 patients who were given chemotherapy at Charing Cross Hospital for GTT between December 1969 and January 1998 (Table 1

**Table 1:**
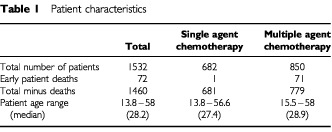
Patient characteristics

). The patients were divided into two groups, one receiving ‘single-agent’ and the other ‘multi-agent’ chemotherapy. Patients in the ‘single agent’ group had low or medium risk disease according to the Charing Cross modification of the WHO GTT scoring system (World Health Organization Scientific Group, 1983) used to stage this disease and received methotrexate and folinic acid (MTX/FA). The ‘multi-agent’ group was composed of those with medium or high-risk disease requiring treatment with either MTX/FA plus actinomycin D or combination drug chemotherapy most frequently consisting of etoposide, methotrexate and actinomycin D (EMA) alternating weekly with cyclophosphamide and vincristine (CO) (Bower and Newlands, 1997). Chemotherapy was completed once hCG levels had fallen to within the normal range and remained there for at least 6 weeks. Patients initially given ‘single-agent’ treatment but whose hCG levels remained elevated (usually because of methotrexate-resistance) then received multi-agent chemotherapy and were included in the ‘multi-agent’ group (Omura, 2000) in this study. Patients who died within weeks of diagnosis or while still receiving treatment were excluded from the study.

The outcome of patients who had become pregnant within 12 months of completing chemotherapy was then reviewed. Their relapse rate was compared to the relapse rate of patients that had not become pregnant within 12 months. Pregnancy and foetal outcomes in both groups were also compared.

### Statistical considerations

Statistical analysis of the results was performed using the Fisher's exact test. The values were considered significant at *P*⩽0.05.

## RESULTS

Of the 1532 patients who were treated for GTT, one patient from the single agent and 71 from the multi-agent chemotherapy groups died either before or during initial treatment and were excluded from further analysis. Of the 1009 patients allocated to single agent (low risk) treatment, 328 then changed to multi-agent chemotherapy because of methotrexate-resistance. Consequently, 681 patients received single-agent chemotherapy and 779 patients received multi-agent treatment. The median ages of the two groups were well matched (Table 1).

Overall, 78 (5.3%) patients relapsed. Of these, 34 were from the single-agent and 44 from the multi-agent group (Table 2

**Table 2:**
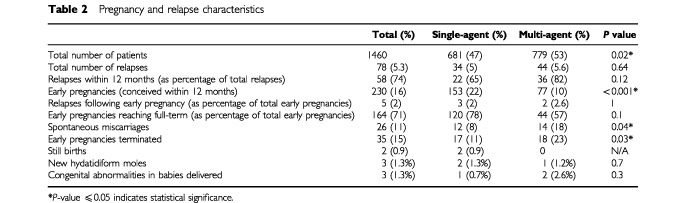
Pregnancy and relapse characteristics

). Most relapses occurred within the first 12 months (65% in the single-agent and 82% in the multi-agent treatment groups).

Despite advice to avoid pregnancy in the first year of follow-up 230 patients (16% of the total) became pregnant within 12 months of completing chemotherapy. Of these, the significant majority were from the single-agent group (67%). Of the 230 patients who became pregnant, three patients within the single agent and two within the multi-agent group relapsed. Therefore, relapses did not occur more frequently in either treatment group. Moreover, the relapse rate appeared to be slightly less in the patients who conceived early than in those women who did not become pregnant in the first 12 months (Table 2).

**Figure 1:**
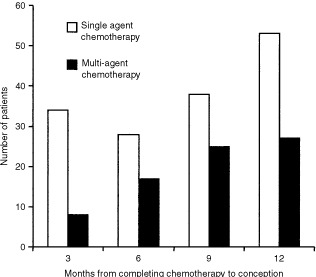
Histogram showing time to conception patients receiving single and multi-agent chemotherapy for GTD.

Interestingly, the pattern of pregnancies differed between the two treatment groups. Though in both treatment groups, more pregnancies occurred in the later 6 months of the follow-up period, this was especially significant in the multi-agent group with 25 out of 77 patients conceiving before 6 months and 52 out of 77 after 6 months (*P*=0.008) (Figure 1

).

**Figure 2:**
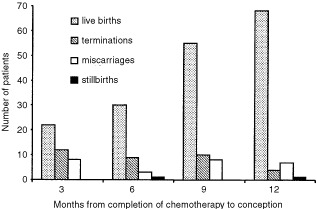
Histogram showing pregnancy outcomes in women who conceived within 12 months of completing chemotherapy.

The majority (71%) of the 230 pregnancies within the 12 month follow-up period resulted in full term live births (Table 2). The time period between completing chemotherapy and conception did not significantly alter eventual pregnancy outcome (Figure 2

). The remaining pregnancies resulted in spontaneous abortions (11%), voluntary terminations (15%), still births (1%) and new hydatidiform moles (1.5%) (Table 2). On comparing both groups there was a significantly higher incidence of terminations and miscarriages in the multi-agent group (Table 2). Of the live births, one baby died after 4 days with no cause of death established. Three babies were born with congenital abnormalities (Hirschprung's disease, fibrosing alveolitis and Down's syndrome). In the five patients who became pregnant and subsequently relapsed, the preceding treatment had been for complete moles. Four of the five women delivered at term but one became breathless at 36 weeks (Table 3

**Table 3:**
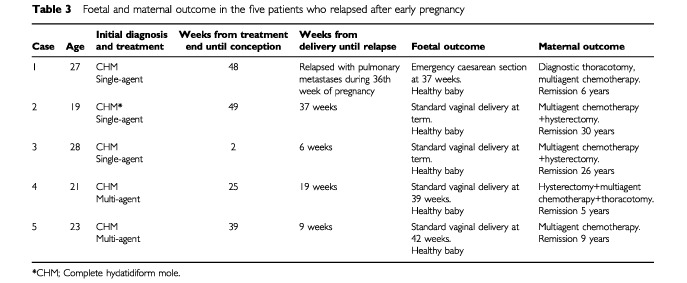
Foetal and maternal outcome in the five patients who relapsed after early pregnancy

, patient 1), was found to have multiple pulmonary metastases and was delivered by Caesarian section. In this case one pulmonary metastasis was resected and histology confirmed choriocarcinoma. Genetic analysis was carried out using fluorescent microsatellite genotyping as previously described (Seckl *et al*, 2000) in order to determine the causative pregnancy. The results shown in Table 4

**Table 4:**
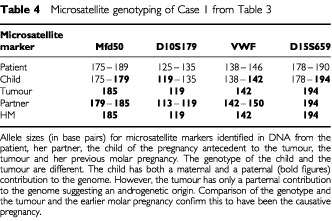
Microsatellite genotyping of Case 1 from Table 3

demonstrate that the choriocarcinoma arose from the preceding complete mole. All five patients were subsequently cured with combination chemotherapy with an additional hysterectomy in one case (median follow-up 9 years range 5–30). Moreover, all their infants thrived (Table 3).

## DISCUSSION

This study aimed to investigate the safety of early conception following chemotherapy for GTT in the single and multi-agent treatment groups. This was achieved by comparing the pregnancy outcomes, rates of disease relapse and development of second tumours to those of other women on our GTT database or in the general population.

### Relapse

Of the 230 patients who conceived in the first 12 months of follow-up only five relapsed. All of these had had a previous complete hydatidiform mole. In one patient we confirmed that the subsequent choriocarcinoma at relapse originated from the previously treated molar pregnancy and was not a new post-term choriocarcinoma. Indeed, in our experience, the preceding hydatidiform mole is usually the cause of subsequent GTT even in the case of several intervening pregnancies (unpublished observations). We therefore believe that the other four cases developed their relapsing GTT from the previous molar pregnancy.

Our results confirmed that relapse is most likely to occur within 12 months of completing chemotherapy. Patients who have received more intensive chemotherapy do not seem to be at a higher risk of disease recurrence than those on single agent treatment. In addition, the incidence of relapse is not increased in patients who become pregnant within 12 months of completing treatment.

### Time to conception and termination rate

There were fewer early pregnancies in the women who had received multi-agent chemotherapy. Of those pregnancies, the majority occurred beyond 6 months from their treatment completion. This is likely to be due to anovulation following more intensive treatment. Studies have shown that the ovary has fewer primordial follicles following chemotherapy (Sieber and Adamson, 1975). Many patients experience a period of anovulation following the EMA/CO treatment regimen. The duration of infertility is usually age-dependent (Kanazawa *et al*, 2000).

Higher risk patients are also less likely to feel physically well after multi-agent chemotherapy and prefer to follow GTT guidelines and delay conceiving. This is another factor contributing to the fewer pregnancies in this group and is also reflected in their significantly greater number of terminations.

By contrast, the low-risk, methotrexate-containing chemotherapy group had a less marked difference in distribution of pregnancies over the 12 months following their chemotherapy. This is because single agent methotrexate causes fewer menstrual irregularities with most patients maintaining a normal menstrual cycle throughout their treatment. Generally, these patients have a greater sense of physical well-being after treatment which contributes to the higher number of conceptions and fewer terminations. Also physicians may be more inclined to institute close monitoring during their pregnancy rather than to advise elective abortion.

### Miscarriages

Patients in the multi-agent group had a significantly higher number of spontaneous miscarriages than those receiving single agent treatment. This again indicates that higher intensity treatment is more likely to have adverse physiological effects. Because hCG monitoring of patients in this study continued after their chemotherapy, a pregnancy would be diagnosed earlier. We would therefore expect higher rates of reported miscarriage than occur in the general population whose hCG levels are not routinely monitored. Thus we compared our results with those of a study of 217 pregnancies in women whose hCG levels were prospectively measured with a similar definition of ‘serological pregnancy’ (Ellish *et al*, 1996). This study recorded a miscarriage rate of 13.7% which compares very favourably with our overall miscarriage rate of 11%. Other studies have reported early miscarriage rates affecting between 8% (Whittaker *et al*, 1983) and 61% (Edmonds *et al*, 1982) of pregnancies. Consequently, though patients in the multi-agent group appeared to be at greater risk of miscarriage (18%) than those in the single agent group (8%), it is unlikely that they are at greater risk than that of the general population.

### Foetal malformation and still birth

Congenital malformations (as defined by the Office for National Statistics (1999)) occurred in 1.3% of the 230 pregnancies conceived within 12 months of chemotherapy. This did not appear to differ from the 1.6% rate recorded in the general population of England and Wales between 1971 and 1998 (Office for National Statistics, 1999). Moreover, our results are in accordance with previous studies showing no correlation between foetal malformation and previous chemotherapy for GTT (Song *et al*, 1988; Tuncer *et al*, 1999a,b; Berkowitz *et al*, 2000). Between 1970 and 1997, the Office for National Statistics recorded an average of 7.08 stillbirths per 1000 pregnancies reported per year in the general population of England and Wales (0.7%) (Office for National Statistics, 1998–1999). Thus, the 1% still birth rate recorded in this study compared favourably with these figures.

### Second hydatidiform mole

In this study, three patients (1.3%) developed new hydatidiform moles, different in histology to their previous invasive GTT and therefore not defined as relapsed or recurrent disease. The risk of second molar disease complicating a subsequent pregnancy has been estimated at around 1% (Vose *et al*, 1988) and in a recent retrospective study of 5030 trophoblastic disease patients there was a recurrent molar pregnancy rate of 0.7% (Lorigan *et al*, 2000). Thus early pregnancy does not appear to increase the chance of having a second molar pregnancy.

### Conclusion

Early pregnancy following chemotherapy for GTT does not increase disease relapse. In terms of foetal outcomes, pregnancy following single agent (low risk) treatment has no effect on rates of miscarriage, still birth or congenital malformation. While early pregnancy after multi-agent treatment does not appear to increase the rate of still birth or congenital malformation, it may transiently reduce fertility and relatively increase the chance of miscarriage. Physicians should still advise patients to avoid pregnancy for 1 year after chemotherapeutic treatment for GTT. This is because, patients are at greatest risk of relapse during this time and the rising hCG of pregnancy can prevent early detection and diagnosis of disease recurrence. This could place both the patient and foetus at risk. However, patients who do become pregnant and are desperate to have a child can be assured of a probable favourable outcome.
